# Home-based oral immunotherapy (OIT) with an intermittent loading protocol in children unlikely to outgrow egg allergy

**DOI:** 10.1186/1710-1492-10-11

**Published:** 2014-02-26

**Authors:** Kyoko Sudo, Shoichiro Taniuchi, Masaya Takahashi, Kazuhiko Soejima, Yasuko Hatano, Keiji Nakano, Tomohiko Shimo, Hayato Koshino, Kazunari Kaneko

**Affiliations:** 1 Department of Pediatrics, Kansai Medical University, 10-15 Fumizono-cho, Moriguchi-shi, Osaka 570-8506, Japan; 2 Brain Inc, Kyoto, Japan

**Keywords:** Egg allergy, Home- based oral immunotherapy, Intermittent loading protocol, Unlikely to outgrow

## Abstract

**Background:**

Home based oral immunotherapy (OIT) for food allergy has often been used for young children in Japan, the majority of whom are believed to outgrow the allergy by the school age, therefore the true efficacy of the therapy has been controversial. The aim of this study was to evaluate the efficacy and safety of a newly developed slow- type home-based oral immunotherapy (OIT) regimen in children with hen’s egg (HE) allergy, who had low likelihood of outgrowing the allergy, with treatment involving only elimination diet.

**Method:**

We retrospectively reviewed the medical records of 43 children with egg allergy (30 males; median age 6) who fulfilled Burks et al.’s criteria of being unlikely to outgrow the allergy. Thirty children who agreed to start OIT were assigned to the treatment group, and 13 who did not want to participate immediately were assigned to the untreated group; the patients underwent an elimination diet for 1 year, during which they were monitored. The OIT regimen involved the intake of the maximum tolerated dose 2 to 3 times a week at home, with initial dose introduction followed by dose build-ups with medical supervision. We statistically evaluated the rate of children who changed their threshold up to 32 g of egg – defined as, oral tolerance induction– in both the groups for 1 year and in the OIT group for 2 years, as well as the rate of children who fulfilled Savage et al.’s criteria of clinical tolerance after reaching the abovementioned remission stage.

**Results:**

The rate of children who achieved oral tolerance induction to 32 g of egg after 1 year in the OIT group (9/30) was significantly higher than that in the untreated group (0/13). The total rate within the OIT group was significantly increased from 9/30 at 1 year to 17/30 at two years without any severe adverse reaction; of the above 17 children, we followed 14 children, and noted that 11 of these were able to obtain clinical tolerance.

**Conclusion:**

The home-based OIT with an intermittent loading protocol was very safe and effective in children with a low likelihood of outgrowing egg allergy.

## Background

Hen’s egg (HE) allergy is one of the most frequent food allergies in Japan, affecting approximately 1 - 5% of young children [1]. Although two- thirds or more of these children are believed to outgrow the allergy by approximately 6 years of age [1-3], some children continue to experience persistent allergic reactions [3-8]. The proportion of children who are not likely to outgrow HE allergy varies in several studies [3,4,6-12]: Savage et al. noted that 81% by age of 4, 45% by 8, 24% by 12, and 9% by 16 still suffer from the persistent HE allergy [3] while Kaneko suggested that these differences result from the varied characteristics of the study population, and indicated that high serum egg- immunoglobulin (-Ig) E level, older age, and the presence of complicated allergy symptoms are predictors of HE allergy persistence [13]. In the present study, we adopted the criteria suggested by Burks et al. [14] to eliminate the children with HE allergy who were likely to outgrow the allergy from our study population. Thereafter, we aimed to elucidate the true efficacy, safety, and convenience of an oral immunotherapy (OIT) regimen through a trial involving an intermittent loading protocol designed to reduce children’s burden while maintaining satisfactory efficacy.

## Methods and patient selection

1) Subjects

The survey was retrospectively performed based on the medical records of 118 children who presented to the day clinic at Kansai Medical University hospital, Osaka, with HE allergy during July 2006 and November 2012. We excluded the following patients from the subjects: the children who exhibited a negative result in an open food challenge test for HE; those who had persistent atopic dermatitis and unstable asthma; those who did not return for a repeat appointment or returned only once after the challenge test; those who fulfilled Burks et al.’s criteria of being likely to outgrow the allergy, i.e., those aged < 5 years, those with a low titers of serum egg white- specific IgE antibody level (<5 UA/ml for children at ≥6 years of age and < 12 UA/ml for children at 5 years of age) [14].

**Figure 1:**
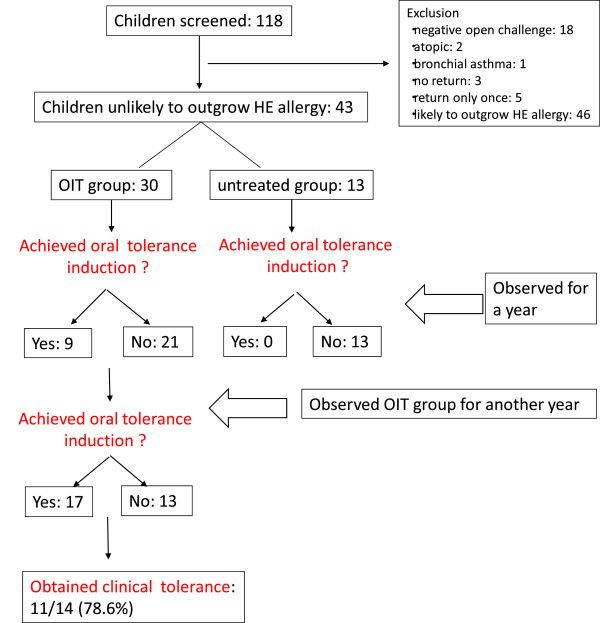
**Study enrollment and outcomes of oral immunotherapy (OIT).** Hen’s egg, HE.

As a result, 75 patients were excluded from the study: negative result in an open food challenge test for HE in 18; persistent atopic dermatitis in 2; unstable asthma in 1; no return for a repeat appointment in 3; return only once after the challenge test in 5; likelihood to outgrow the allergy in 46 (Figure 1).

Thus, the data for statistical analysis was based on the clinical outcomes of 43 children (median age, 6 years [range, 5-12 years], male/female, 30/13), including 30 children who agreed to follow the OIT regimen, and therefore were assigned to the treatment group, and 13 who did not want to participate immediately and were assigned to the untreated group. For ethical reasons, this allocation was not performed in a random manner and was based on the children’s and parents’ decision. The baseline characteristics of these subjects are shown in Table 1; no significant differences were noted between the 2 groups, except for “skin symptoms during challenge test”.

2) Oral food challenge test

**Table 1:** Baseline characteristics of patients in the study group

**Characteristic**	**Group**		***P *** **value**
	**OIT (N = 30)**	**Untreated (N = 13)**	
Age at the challenge test			
Median	6.00	7.00	0.967
Range	5.00 - 12.00	5.00 - 8.00	
Initial total IgE antibody level (UA/ml)			
Median	905.5	618.9	0.272
Range	108.5 - 9978.0	82.8 - 2054.0	
Initial egg-specific IgE antibody level (UA/ml)			
Median	17.95	17.80	0.905
Range	5.25 - 100.00	5.23 - 100.00	
Duration since last episode (years)			
Median	2.00	2.00	0.807
Range	0.00 - 6.00	0.50 - 6.00	
Condition of elimination -*			
Partial	6.7	7.7	1.000
Complete	93.3	92.3	
Presence of other food allergies -*			
No	13.3	30.8	0.217
Yes	86.7	69.2	
Frequency of allergic symptoms at accidental ingestion -*			
Never	20.0	30.8	0.104
Once	13.3	30.8	
2-10 times	60.0	38.5	
>10 times	6.7	0.0	
Grade of anaphylaxis at accidental ingestion -*			
1	73.3	69.2	0.893
2	20.0	30.8	
3	0.0	0.0	
4	6.7	0.0	
Frequency of emergency visit at accidental ingestion -*			
Never	83.3	92.3	0.482
Once	10.0	0.0	
2-5 times	6.7	7.7	
Grade of anaphylaxis at challenge test -*			
1	46.7	53.8	0.207
2	20.0	46.2	
3	33.3	0.0	
4	0.0	0.0	
Symptoms during the challenge test -*			
Respiratory: cough, asthma, difficulty in breathing	36.7	38.5	1.000
Skin: rash, hives, eczema	60.0	23.1	0.045
Gastrointestinal: vomiting, diarrhea, abdominal pain	26.7	23.1	1.000
Dislike of eggs -*			
No	90.0	100	0.542
Yes	10.0	0.0	

*Percentage within the group.

All egg-food challenges were open challenges, and were performed in hospital settings and supervised by physicians. Clinical features of a reaction to HE were investigated for clinical purposes via an open challenge test as described in our previous study [15]. Briefly, a double-blind placebo-control food challenge is the gold standard for clinical studies, but is a time-consuming test for general practice. We could not assess the subjective symptoms by the open challenge test. Therefore, if the patients had subjective allergic symptoms such as nausea, abdominal pain, sore throat, or itching, we increased the loading dose before the objective symptoms appeared. During the challenge, full emergency equipment was at hand. The children’s parents provided informed consents prior to enrollment in the study. Patients taking anti-histamines were asked to avoid them for at least 72 h before the challenge, but topical steroids were allowed. Patients were admitted to our day clinic in the morning in a fasting state. Challenge material for open challenges was cooked egg (an omelet baked by using a Japanese rectangular fry pan), and the omelet was further steamed for 10 minutes in the purpose of heating egg-white protein completely. The initial challenge dose and the following doses were customized according to the history of the last reaction, but were similar in most patients (omelet: 1, 2, 4, 8, 16, and 32 g). When the patients tolerated the first dose, the following one was given every 30 min. When a reaction to a very low dose was suspected, the first challenge dose was 0.5 g. The challenge was interrupted if children demonstrated unambiguous clinical reactivity or after the administration of 63 g of egg. All children were then observed for at least 3 more hours after the end of the feeding. If a child exhibited obvious allergic symptoms, such as rash, coughing, vomiting, or diarrhea to HE under loading doses of less than 32 g, he/she was considered to have positivity to HE. Otherwise, they were considered negative.

3) Home-based OIT with an intermittent loading dose

All the subjects were outpatients. Cooked egg which was used in the preliminary open challenge test was prepared in the same manner as the material administered for OIT [16]. A medium- sized whole egg was used to make a rectangular omelet – one of the most popular food preparations in Japan – that weighed approximately 64 g and could be cut into half with ease. The directed dose – either 1, 2, 4, 8, or 16 g – was easy to prepare consistently during the study. The omelet was further steamed for 10 minutes in order to heat egg white completely. The initial dose of OIT was set at a sub-threshold dose, which was usually half or one- fourth of the threshold dose determined during a preliminary open challenge test. For example, if the threshold dose was 2 g, the child would begin with 1 g or 0.5 g, according to the severity of the symptoms presented in the preliminary challenge test. In cases where no adverse reactions were noted, the initial dose was administered intermittently at least twice a week at home for 2 months as a maintenance dose. The children were challenged to ingest a double dose under medical supervision at every 2-month follow-up visit. The escalation phase was repeated in the same manner until the child was able to ingest 32 g of the cooked egg, the content of which is approximately half of a medium- sized HE – this was defined as the minimum dose required to achieve oral tolerance induction to one of the most popular Japanese food preparations. The children were encouraged to continue ingesting a further increased dose as a less well-cooked omelet (for example, omelet without steaming and soft-cooked) even after achieving the remission stage.

4) Laboratory tests

Blood samples were collected before starting home-based OIT, during the remission stage, and at the stage that the child achieved ad libitum egg consumption.

5) Analyses

Statistical analyses of 2 aspects were performed: [1] the rate of oral tolerance induction (defined as achieving an intake of 32 g of well-cooked omelet without any reaction) in the OIT and control groups at 1 year; [2] the same rate in the OIT group at 1 year and at 2 years. We also analyzed the number of children who obtained clinical tolerance after oral tolerance induction after 2 years of OIT, according to Savage et al.’s most inclusive definition – tolerating ad libitum egg consumption including raw egg with no adverse reaction in the past 12 months, and egg IgE levels of <6 UA/ml [3]. Furthermore, we statistically analyzed the same factors indicated in Table 1 among the children in the OIT group, to determine whether the baseline characteristics differed between the subjects who achieved oral tolerance induction or clinical tolerance to a whole HE and those who failed to do so.

6) Statistics

We statistically evaluated the clinical outcome of the OIT group (30 children) and the untreated group (13 children) at 1 year, at a 2-sided alpha level of 0.05, to detect a significant difference between the 2 groups. Baseline characteristics of the patients were tested by using Fisher’s exact test and the Mann-Whitney U test. Fisher’s exact test was used to evaluate between-group differences with regard to achieving oral tolerance induction by 1 year. We also evaluated the differences in achieving the abovementioned remission stage by 1 year and by 2 years within the OIT group by using the sign test. All analyses were performed with IBM SPSS Statistics, version 11.0 (SPSS Inc. Chicago, IL, USA).

7) Ethical issue

The ethics review board of Kansai Medical University approved the study (#1210), and informed consent was obtained from the parent of each child.

## Results

### Clinical outcomes

**Figure 2:**
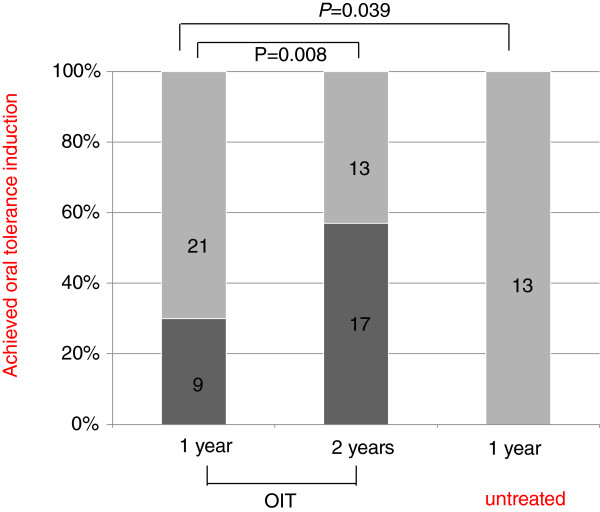
**The rate of children with a change in the threshold to 32 g of HE – defined as oral tolerance induction – at 1 year and 2 years in the oral immunotherapy (OIT) and untreated groups.** The abovementioned remission rate at 1 year in the OIT group (30.0%) was significantly higher than that of the untreated group (0.0%). Moreover, the remission rate at 2 years in the OIT group (56.7%) was significantly increased from that at 1 year (Fisher’s exact test). Dark gray square: oral tolerance induction to 32 g of HE; light gray square: remission not yet achieved.

Study enrollment and outcomes are shown in Figure 1. Nine of 30 children (30%), and 0/13 (0%) children successfully achieved oral tolerance induction by 1 year in the OIT group and untreated group, respectively; there was a statistically significant difference between the 2 groups, as shown in Figure 2. Some children who did not achieve the abovementioned remission stage by 1 year achieved one by 2 years, and the total rate within the OIT group was significantly increased to 17/30 (56.6%) by 2 years (Figure 2). The 9 children who achieved oral tolerance induction by 1 year were among those 17 children. The period required for those 17 children to reach the abovementioned phase was 75 – 726 days (median, 351 days). The levels of egg white-specific IgE decreased significantly in both the groups; however, the OIT group exhibited a greater decrease as compared with the untreated group (median egg white-specific IgE level, 17.0 -5.3 vs. 17.8 - 12.7 UA/ml, respectively). Four children took more than 2 years to reach the remission stage, and we did not include them in the statistical analysis. We further followed the 17 children who had achieved oral tolerance induction for > 1 year to assess whether they could achieve clinical tolerance to whole HE according to the definition described earlier. Three of these children were excluded at the point of termination of the study; one patient whose egg IgE level was unknown because he refused to have a blood sample taken, although he achieved ad libitum egg consumption, based on the clinical criteria; one patient for whom oral tolerance induction was achieved within 1 year of study termination, and who was not observed for the entire 12-month period; and one patient in whom OIT was discontinued as inflammatory bowel disease suddenly developed after accidental ingestion of a milk product to which he was severely allergic. Thus, we followed 14 children after the remission stage and 11 (78.6%) of these children obtained clinical tolerance. The duration from oral tolerance induction to the ad libitum consumption of any form of egg for >1 year in the patients was 366 – 988 days (median, 818 days).

### Baseline characteristics in OIT group between remission and failure groups

Although there were no significant differences between the subjects who had achieved oral tolerance induction and those who had failed to do so, there were several significant differences between the subjects who had obtained stabilized clinical tolerance and those had not. Factors such as initial egg white-specific IgE level (*p* = 0.012), grade of anaphylaxis at the challenge test (*p* = 0.020), and respiratory symptoms during the challenge test (*p* = 0.023) showed significant differences, as shown in Table 2.

**Table 2:** Differences in baseline characteristics between the subjects who obtained clinical tolerance and those who failed

**Characteristic**	**Group**		***P *** **value**
	**Tolerance (N = 11)**	**Failure (N = 19)**	
Age at challenge test			
Median	6.00	6.00	0.081
Range	6.00 - 12.00	5.00 - 11.00	
Initial total IgE antibody level - UA/ml			
Median	668.0	997.0	0.378
Range	108.5 - 3801.0	161.5 - 9978.0	
Initial egg-specific IgE antibody level - UA/ml			
Median	14.60	25.50	0.012
Range	5.25 - 27.80	5.33 - 100.00	
Duration since Last Episode - yr			
Median	2.00	2.00	0.758
Range	0.50 - 6.00	0.00 - 6.00	
Condition of elimination*			
Partial	9.1	5.3	1.000
Complete	90.9	94.7	
Having other food allergies*			
No	18.2	10.5	0.611
Yes	81.8	89.5	
Frequency of allergic symptoms at accidental ingestion*			
Never	27.3	15.8	0.807
Once	9.1	15.8	
2-10 times	54.5	63.2	
>10 times	9.1	5.3	
Grade of anaphylaxis at accidental ingestion*			
1	90.9	63.2	0.095
2	9.1	26.3	
3	0.0	0.0	
4	0.0	10.5	
Frequency of emergency visit at accidental ingestion*			
Never	100	73.7	0.068
Once	0.0	15.8	
2-5 times	0.0	10.5	
Grade of anaphylaxis at challenge test*			
1	72.7	31.6	0.020
2	18.2	21.1	
3	9.1	47.4	
4	0.0	0.0	
Symptoms during challenge test*			
Respiratory: cough, asthma, difficulty in breathing	9.1	52.6	0.023
Skin: rash, hives, eczema	54.5	63.2	0.712
Gastrointestinal: vomiting, diarrhea, abdominal pain	27.3	26.3	1.000
Dislike of Eggs*			
No	90.9	89.5	1.000
Yes	9.1	10.5	

*percentage within the group.

### Safety data during the OIT period

Sixteen of 30 (53.3%) children in the OIT group experienced adverse reactions following challenges involving dose build-ups in the hospital, and the same rate was noted for home OIT intake. Some children experienced accidental ingestion of offending foods other than HE, but we did not include the events that were not related to HE in the analyses.

Table 3 shows the percentage of the adverse events that occurred for a trial in each person either at home or at the outpatient clinic. There were 31 adverse events among the 477 challenges involving dose build-ups in the hospital (6.5%); according to Sampson’s classification [17], there were 25 (80.6%) grade 1 reactions and 6 (19.4%) grade 2, and none of the children experienced grade 3 or 4. The associated symptoms included oral and pharyngeal (17.1%), skin (20.0%), respiratory (20.0%), gastrointestinal (37.1%), and other (5.7%) symptoms. For treatment of these symptoms, 11/16 (68.8%) of the children received medication, including oral anti-histamines (54.8%), topical steroids (6.5%), and nebulized beta-2 agonist (16.1%); none of the children received treatment with epinephrine injection.

**Table 3:** Adverse events and treatments during oral immunotherapy (OIT)

**Situation**		**Symptom type**				
	**Total frequency**	**Oral**	**GI**	**Skin**	**Resp.**	**Other**
	*times per trial per person* * × 100(%)					
At home	0.485	0.091	0.262	0.100	0.079	0.081
With dose build-up	9.117	1.150	3.450	3.583	1.283	2.350
**Situation**	**Treatment**					
	**Oral anti-histamine**		**Oral betamethasone**	**Topical steroid**	**Nebulized β**_**2 **_**stimulant**	**Intravenous prednisone**
	*times per trial per person* * × 100(%)					
At home	0.360		0.058	0.010	0.015	0.005
With dose build-up	4.183		0.000	2.433	0.983	0.000

*The number of adverse reactions was divided by the number of trials of each patient, and then an average value for the 30 patients was calculated.

The OIT duration of all patients was 0.50 - 5.25 years (median, 2.54 years); trials at home, 65 - 685 times (median, 331 times); trials with dose build-up, 3 - 32 times (median, 15 times).

GI, gastrointestinal: abdominal pain, vomiting, diarrhea.

Resp., respiratory: cough, wheeze, asthma, difficulty in breathing.

During the maintenance phase at home, there were 45 adverse events among 10380 trials (0.43%). There were 34 (75.6%) grade 1, 10 (22.2%) grade 2, and 1 (2.2%) grade 3 reactions, and none of the children experienced grade 4 reaction. The child who experienced a grade 3 adverse reaction had an exercise- induced anaphylaxis and was admitted to the emergency room. The associated symptoms in these patients included oral and pharyngeal (18.1%), skin (18.1%), respiratory (18.1%), and gastrointestinal (45.5%) symptoms. For the treatment of these symptoms, 16/16 (100.0%) of the children received medication, including oral anti-histamines (75.6%), oral betamethasone (8.9%), topical steroids (2.2%), nebulized beta-2 agonist (4.4%), and intravenous prednisone (2.2%); none of the children received treatment with epinephrine injection.

## Discussion

The efficacy of various types of OIT for children with egg allergy has recently been reported with favorable outcomes [14,18-21], though only few studies have been performed by placebo-control randomized manner [21,22]. One of the characteristics in our study was to select a population with a low likelihood of outgrowing the egg allergy including an untreated group as we attempted to indicate the efficacy of our regimen more strictly. A further characteristic of our study was the long duration of follow-up, which facilitated the assessment of whether the children could achieve not only temporal remission but also clinically stabilized oral tolerance. It has been suggested that for children with moderate to severe egg allergy, a longer period (3 – 5 years) and/or a higher dose loading might be beneficial [21,22]. Indeed, in our study, some children who did not achieve oral tolerance induction by 1 year were able to achieve one by 2 years, and the achievement rate increased by nearly double (the number of patients who achieved temporal remission by 1 year: 9, those who achieved temporal remission by 2 years: 17). In addition, it should be noted that the longest period required to achieve oral tolerance induction among these 17 children was 726 days and 11 out of 14 patients (78.6%) who reached the abovementioned remission stage within 2 years obtained clinical tolerance. Thus, a long-term observation of at least two years is needed to accurately determine the efficacy of slow- type OIT, in particular, in children with a low likelihood of outgrowing egg allergy.

We analyzed the baseline characteristics between the subjects who achieved oral tolerance induction or clinical tolerance and those who failed to do so. Factors such as the initial egg white-specific IgE level, grade of anaphylaxis at the challenge test, and respiratory symptoms during the challenge test showed significant differences between the subjects who obtained clinical tolerance and those who failed to do so. Interestingly, in an OIT study for milk allergy, Vázquez-Ortiz et al. reported that pre-existing asthma was associated with a group with persistent reactions [23]. However, the underlying mechanism explaining the relationship between asthma and difficulties in OIT is unclear.

Most previous standardized protocols involve multiple dose increasing phase at an initial day or several days, termed as a rush escalation phase, which is either performed in an outpatient clinic or in a hospital, followed by a more gradual weekly to biweekly dose escalation period at home [1,12,24-27]. Although this approach greatly contributes to desensitization and potential permanent tolerance for patients with food allergies, adverse events occur mainly during the rush escalation phase. Recently, new trials using protocols that omit a rush escalation phase known as home-based OIT, have been used due to the improved safety associated with these methods. Our regimen also omits the rush escalation phase, and is unique in that we used an intermittent loading dose (i.e., 2 to 3 times a week) instead of daily loading as in previous home-based OITs [14,18-20]. In order to ensure safety, we also kept a fixed dose throughout the maintenance period at home, and avoided any escalations, as indicated in previous protocols [14,18-20]. The advantage of intermittent loading is the safety demonstrated by the number of adverse events related to doses administered at home; 45/10380 (0.43%) for home-based OIT throughout the current study period, which is considerably better than that of the previous studies ranging from 0.99% to 24.2% [14,18,20,28]. This is due to an opportunity to omit the loadings on weekends, or sick days; the former lower the risk of developing allergic reaction out of office hours of the clinic and the latter is associated with the increased risk of allergic reaction. Furthermore, the children do not have to experience the daily discomfort of ingesting undesirable food, which would also facilitate better compliance for a long duration of the OIT period.

There are several limitations in our study. First, it was retrospective and not randomized. The observational period of the untreated group was limited to 1 year, which could not be extended because all the patients wanted to start either the slow-type home-based OIT or rush-type OIT after the 1-year observation. Therefore, we could not maintain a control group for the entire duration of the study. Second, the immunological markers assayed were limited to the IgE level, and we were not able to include the IgG4 levels or skin prick test. Finally, the most important limitation was the absence of a double blind-placebo food challenge test after several weeks of complete withdrawal of egg products as the tolerance judgment test, although we adopted the criteria suggested by Savage et al. [3].

## Conclusions

Our home-based OIT with an intermittent loading protocol is safe and rather effective for children with a low likelihood of outgrowing the egg allergy. Thus, intermittent loading protocol is a novel approach that expands the possibility of an active treatment to improve the quality of life of patients and their families.

## Competing interests

All authors have declared that they have no conflicts of interests.

## Authors’ contributions

Conception and design, KS and ST; acquisition of data, KS, ST, MT, KS, YH, KN and TS; analysis and interpretation of data, KS, ST and HK; drafting of the manuscript, KS and HK; critical revision, KS, ST and HK; statistical analysis, HK; and supervision, ST and KK. All authors read and approved the final manuscript.
